# The Standard Dictionary of Funk & Wagnalls

**Published:** 1896-01

**Authors:** 


### The Standard Dictionary of Funk & Wagnalls

So frequently and so favorably noticed in these pages, has become the victim of a most remarkable assault upon its standing at the hands of malignant enemies. This defamation is nothing more or less than the selection from the pages of this dictionary of eighteen words of an indecent character, and publishing them together with their definitions in a circular which is being sent to teachers, parents, and guardians, as an argument against the purchase of the dictionary for family and school use. It matters little that these same words are found in other dictionaries: that other words of similar character were rejected by the builders of the *Standard*, and therefore not suffered to appear, the defamers nevertheless continue these shameful assaults obviously with a view to limiting sales.

These people, their names and connections, as well as their motives, should be known and execrated.

The Funk & Wagnalls Company are to be congratulated from one point of view, and that is: Slander ever loves a shining mark.

The extraordinary character of the attack is a brilliant testimonial to the success of this dictionary. The envious would not go to such length to check it, if it were not making great progress. As "persecution is the seed of the church" the publishers of the *Standard* may look for even greater success with their magnificent book.

